# Are Small Molecules Effective in Treating Inflammatory Pouch Disorders Following Ileal Pouch-Anal Anastomosis for Ulcerative Colitis? Here Is Where We Stand

**DOI:** 10.3390/biom14091164

**Published:** 2024-09-17

**Authors:** Antonietta Gerarda Gravina, Raffaele Pellegrino, Giovanna Palladino, Giuseppe Imperio, Francesco Calabrese, Andrea Pasta, Edoardo Giovanni Giannini, Alessandro Federico, Giorgia Bodini

**Affiliations:** 1 Hepatogastroenterology Division, Department of Precision Medicine, University of Campania Luigi Vanvitelli, Via L. de Crecchio, 80138 Naples, Italy; 2 Gastroenterology Division, Department of Internal Medicine, Istituto di Ricovero e Cura a Carattere Scientifico, Policlinico San Martino, University of Genoa, Viale Benedetto XV, 16132 Genoa, Italy

**Keywords:** ulcerative colitis, pouchitis, chronic pouchitis, chronic antibiotic-refractory pouchitis, chronic antibiotic-dependent pouchitis, Crohn’s disease of the pouch, ileal pouch-anal anastomosis

## Abstract

Ulcerative colitis (UC) management encompasses conventional and advanced treatments, including biological therapy and small molecules. Surgery, particularly in the form of ileal pouch-anal anastomosis (IPAA), is indicated in cases of refractory/severe disease. IPAA can lead to acute complications (e.g., acute pouchitis) as well as late complications, including chronic inflammatory disorders of the pouch. Chronic pouchitis, including the antibiotic-dependent (CADP) and antibiotic-refractory (CARP) forms, represents a significant and current therapeutic challenge due to the substantial need for evidence regarding viable treatment options. Biological therapies have shown promising results, with infliximab, adalimumab, ustekinumab, and vedolizumab demonstrating some efficacy in chronic pouchitis; however, robust randomized clinical trials are only available for vedolizumab. This narrative review focuses on the evidence concerning small molecules in chronic pouchitis, specifically Janus kinase (JAK) inhibitors and sphingosine-1-phosphate receptor (S1P-R) modulators. According to the preliminary studies and reports, Tofacitinib shows a potential effectiveness in CARP. Upadacitinib presents variable outcomes from the case series, necessitating further evaluation. Filgotinib and ozanimod demonstrate anecdotal efficacy. This review underscores the need for high-quality studies and real-world registries to develop robust guidelines for advanced therapies in post-IPAA inflammatory disorders, supported by vigilant clinical monitoring and ongoing education from international IBD specialist societies.

## 1. Introduction

Ulcerative colitis (UC) is an inflammatory bowel disease (IBD) that currently necessitates a complex therapeutic management commensurate with the severity of the condition. This management encompasses conventional treatments primarily relying on 5-aminosalicylic acid (5-ASA) derivatives administered orally or topically, topical and systemic steroids, traditional immunosuppressants (such as azathioprine), and advanced therapy involving biologics and small molecules [1]. Nevertheless, in the case of acute severe disease, patients who are not responsive to intravenous steroids or rescue therapy (i.e., infliximab or cyclosporin), or in the case of dysplasia/colorectal cancer, surgical treatment is indicated. [2]. Among these procedures, restorative proctocolectomy with the construction of an ileal pouch-anal anastomosis (IPAA) stands out as the pillar surgical approach [2]. Among the prevalent late complications associated with IPAA, chronic inflammatory disorders of the pouch, such as chronic pouchitis and Crohn’s-like disease of the pouch, take a prominent stance [2]. The pathogenesis of pouchitis is prevalently inflammatory. Histological features are characterized by ulcerations, the infiltration of neutrophilic leukocytes, and crypt abscesses, which are typical of an acute process. Furthermore, the blunting of the villi, hyperplasia of the crypt cells, and increased mononuclear cells in the lamina propria could be identified, representing signs of a chronic inflammatory process [3].

The IPAA assumes distinct anatomical configurations depending on the surgical technique employed, specifically concerning the ileal arrangement crafted by the surgeon to construct the neo-reservoir. It encompasses three primary pouch forms—the J-pouch, S-pouch, and W-pouch [4]. Among these, the J-pouch is widely favored for its ease of construction and commendable functionality [4]. Establishing a new non-rectal reservoir necessitates a series of adaptive changes in the defecatory function of the patient. This function distinctly differs from that of the normal colon and entails control over the frequency of bowel movements, a process that may extend over several months following the creation of the IPAA [5]. In addition, a neoplastic risk is inherent to the pouch, as substantiated by comprehensive studies, indicating an incidence of pouch neoplasia ranging between 1.3% and 6.9% at 10 and 20 years following its construction [6,7].

This underlines the importance of endoscopic and histological monitoring of the pouch. Moreover, pouch dysplasia has been found to occur more frequently in patients with pre-operative colorectal neoplasia [8] and those with long-standing chronic inflammatory disorders of the pouch [9].

Moreover, chronic pouchitis is pragmatically differentiated into chronic antibiotic-dependent pouchitis (CAPD) and chronic antibiotic-refractory pouchitis (CARP) based on either dependence (recurrence of pouchitis shortly after discontinuation of antibiotic therapy within days or a few weeks of cessation) or refractoriness to first-line antibiotic therapy [10].

The treatment of CADP involves chronic antibiotic therapy at the minimum effective dose, typically utilizing fluoroquinolones such as ciprofloxacin intermittently with periods of discontinuation [10]. Alternatively, a cyclic approach involves rotating between antimicrobial mechanisms, such as ciprofloxacin, vancomycin, and metronidazole [10]. However, this approach does not always prevent pouchitis recurrence, and the criteria for CARP may be met. Therefore, it is necessary to supplement with immunosuppressive therapies. The most recent guidelines recommend employing therapies already approved for UC or Crohn’s disease in such cases [10].

As of now, the “EARNEST” trial stands as the sole robust study, providing evidence of efficacy compared to the placebo for one of the therapies already approved for IBD (i.e., vedolizumab) in the treatment of chronic pouchitis in patients with IPAA following UC [11]. Consequently, vedolizumab (an α_4_β_7_ integrin inhibitor [12]) is currently the only advanced medical treatment approved by the FDA for this indication [10].

An additional small, randomized trial involving just over ten patients with adalimumab failed to demonstrate a clear clinical benefit [13].

Unfortunately, there is a limited availability of randomized controlled trials assessing the efficacy of already approved advanced therapies, aside from the two studies mentioned. Consequently, there is a pressing need to vigilantly monitor the available evidence regarding all approved medications for IBD concerning their potential therapeutic impact on chronic pouchitis. If not adequately controlled, this condition can often lead to pouch failure, necessitating the creation of a permanent ileostomy [14].

Among the currently approved therapies for UC are Janus kinases (JAK) inhibitors (such as tofacitinib [15], filgotinib [16], and upadacitinib [17]) and sphingosine-1-phosphate receptor (S1P-R) modulators (like ozanimod [18] and etrasimod [19]). Within the selectivity profiles of JAK inhibitors, tofacitinib inhibits a significant portion of kinases in this family (i.e., JAK1, JAK2, JAK3, and JAK-related tyrosine kinase-2) and is therefore identified as a “pan-JAK inhibitor”, while upadacitinib and filgotinib primarily act as selective JAK1 inhibitors [20]. This narrative review aims to examine and gather all the available evidence on the therapeutic potential of these two molecules (collectively called “small molecules”) in treating chronic inflammatory disorders of the pouch in patients undergoing IPAA for UC.

## 2. Review Design

The exploration for studies congruent with the aims of this review was carried out across three principal databases—MEDLINE, EMBASE, and Web of Science. The designated search criteria for each database were as follows:

- MEDLINE: (pouch OR pouchitis OR IPAA OR ileal pouch-anal anastomosis) AND (tofacitinib OR filgotinib OR upadacitinib OR ozanimod OR etrasimod);
- EMBASE: (‘pouch’ OR ‘pouchitis’ OR ‘IPAA’ OR ‘ileal pouch-anal anastomosis’) AND (‘tofacitinib’ OR ‘filgotinib’ OR ‘upadacitinib’ OR ‘ozanimod’ OR ‘etrasimod’);
- Web of Science: ((ALL = (pouch) OR ALL = (pouchitis) OR ALL = (IPAA) OR ALL = (ileal pouch-anal anastomosis)) AND (ALL = (tofacitinib) OR ALL = (filgotinib) OR ALL = (upadacitinib) OR ALL = (ozanimod) OR ALL = (etrasimod)).

In the aforementioned search engines, no specific filters were applied that could select for particular study types or specific language filters. Given this review’s narrative and non-systematic nature, the specific criteria for including or excluding the studies used to prepare this work were not predefined. We did not specify precise time frames for the databases searched until this review’s latest update window (16 August 2024).

## 3. Small Molecules in Inflammatory Disorders of the Pouch: Why?

Although a precise and linear mechanism through which small molecules might precisely determine their potential benefits in pouch inflammatory disorders is not fully delineated, several considerations can be postulated based on the evidence already available.

Various pathogenic hypotheses have enriched the pathogenesis of inflammatory disorders of the pouch post-IPAA. These include immune alterations, genetic susceptibility, microbiota-related factors (such as the stasis of fecal material accompanied by bacterial overgrowth and consequent dysbiosis with a reduction in short-chain fatty acids), as well as surgery-related factors like ischemia-reperfusion phenomena and the generation of reactive oxygen species [21]. Nonetheless, the role of *Clostridioides difficile* as a trigger for such disorders is also well recognized [22].

In the context of immune dysfunction, which is particularly relevant to our discussion, it has been hypothesized that, in pouchitis, there are upregulated regulatory mechanisms of the intracellular signaling pathways mediated by toll-like receptors (TLRs) in response to chronic stimulation by bacterial-derived products [23]. Indeed, there appears to be an increased expression of TLR4 in the ileal pouch mucosa of patients with UC [23]. A malfunction of the TLR system leads to an inadequately controlled activation of nuclear factor kappa-light-chain-enhancer of activated B cells (NF-κB), a critical transcription factor in IBD [24]. This promotes an imbalance in the transcription of pro-inflammatory genes, regulating a wide range of pro-inflammatory molecules, most notably tumor necrosis factor (TNF) and interleukins (IL)-1, 2, 3, and 12 [21,25,26]. A significant portion of IBD pharmacology has been built around blocking the pathways induced by NF-κB, with anti-TNF agents being among the first and most prominent therapies [27,28].

The NF-κB transcription factor channels many pro-inflammatory actions through the JAK-STAT pathway, which can act as a precise upstream regulator of NF-κB itself [29]. Nonetheless, inhibition of the JAK-STAT system mediated by JAK inhibitors can also directly inhibit the production of pro-inflammatory cytokines, including those previously mentioned that are upregulated in inflammatory disorders of the pouch [20].

It is also known that S1P signaling activates various pro-inflammatory pathways, such as the NF-κB/IL-6/STAT3 pathway, leading to the increased production of pro-inflammatory cytokines, including IL-6 [30].

Consequently, small molecule-mediated inhibition of the JAK-STAT and SP1 pathways could reduce the inflammatory burden in chronic pouch inflammatory disorders by decreasing the pro-inflammatory load within its molecular microenvironment.

## 4. JAK Inhibitors

### 4.1. Tofacitinib

To date, tofacitinib represents the small molecule with the most extensive body of evidence available, as well as the one that, based on the data from the literature, appears to have been more frequently employed in patients with inflammatory disorders of the pouch post-IPAA for UC. Nevertheless, the evidence is derived from non-randomized controlled studies.

A multicentric retrospective observational study was conducted by the “Groupe d’Étude Thérapeutique des Affections Inflammatoires du Tube Digestif” (i.e., GETAID) registry to assess steroid-free remission eight weeks after treatment (defined as modified pouchitis disease activity index, mPDAI < 5) with tofacitinib in twelve patients, predominantly females, with a median age of 38 years [31]. Four patients achieved complete remission, while half showed a clinical response (a reduction in mPDAI of at least two points compared to baseline). Treatment persistence was evaluated up to a median follow-up of 25.9 months, with only four patients maintaining treatment. Many patients had previously received other biologics before tofacitinib (66.7% with vedolizumab and 41.7% with ustekinumab), with a median of 2.5 prior to the advanced treatments before starting tofacitinib. The safety profile was assessed with adverse events in four patients, necessitating discontinuation in one case due to herpes zoster and in another case due to severe fatigue.

Ribaldone et al. [32] also described a case series, including eight patients treated with tofacitinib for CARP, with a median age of 42.75 and a median time from IPAA creation of 10.37 years. All the included patients had undergone at least one treatment with another advanced therapy for CARP before initiating tofacitinib (mostly vedolizumab or anti-TNF), and six of these achieved a steroid- and antibiotic-free clinical response at three months (defined as a reduction of at least one point from the baseline of the mPDAI), while four achieved a genuine steroid- and antibiotic-free clinical remission.

Another U.S. case series evaluated the clinical response to tofacitinib (assessed as a reduction of at least two points from the baseline PDAI at 3 and 12 months from the start of treatment) in 14 cases, predominantly males, with chronic pouchitis or Crohn’s-like disease of the pouch (the latter representing 79% of the sample), with a median age of 44 years [33]. One case had a previous diagnosis of indeterminate colitis before IPAA, and the median time frame from IPAA creation in the overall sample was 16 years. The median duration of tofacitinib therapy was 12 months, with pouch failure occurring in 29% of the patients. Three of these patients achieved clinical response at three months, and another three achieved it subsequently. The safety profile in this series was favorable, with no serious adverse events reported.

A final small open-label study conducted by Syal et al. [34] enrolled six patients with chronic pouchitis, of whom 67% had previously been exposed to anti-TNF agents and 50% to other biologics (i.e., vedolizumab and ustekinumab). They were all treated with a standard induction regimen of 10 mg twice daily for eight weeks, with a clinical response rate of 67% and a clinical remission rate of 50% (both calculated based on an mPDAI score <5 for remission and decreased by at least two points compared to baseline for response). The safety profile in this study included only one serious adverse event, a case of sepsis due to *Shigella* infection, with tofacitinib reinitiating following antibiotic treatment.

In an abstract presented at the ECCO 2024 congress, Khoo et al. [35] finally reported the clinical and endoscopic response rates of 55% and 58%, respectively, in a total of 33 patients with chronic pouchitis treated with tofacitinib after eight weeks at the standard induction dose as per the UC datasheet.

Within the domain of isolated case reports on tofacitinib’s use in inflammatory pouch disorders, a notable case involves a 20-year-old woman diagnosed with ulcerative colitis at the age of 14 [36]. Despite the optimal medication and surgery at 18, she developed CARP. Conventional treatments, anti-TNF-α therapy, steroids, and vedolizumab yielded unsatisfactory results. Tofacitinib therapy demonstrated symptom improvement and avoided the need for surgery. The described patient experienced a rapid reduction in PDAI from 10 to 3 shortly after the initiation of therapy. An endoscopic response was also observed at six months, with a maximum treatment persistence reported in the seven-month follow-up period.

Another case involves a 48-year-old male with CARP previously treated with infliximab for a decade (also with therapeutic optimization every six weeks) and azathioprine, along with local infliximab injections [37]. The patient faced a significant issue of iron deficiency anemia. Tofacitinib treatment led to the resolution of anemia in a five-month follow-up, rapid clinical improvement, and a reported treatment persistence of one year.

However, another case involves a 68-year-old individual who underwent IPAA four years earlier, experiencing a failure with ustekinumab [38]. Tofacitinib, initially administered at 20 mg/day, effectively controlled increased evacuation frequency. Upon dosage reduction to a maintenance dose (10 mg/day), therapeutic optimization was achieved at the initial dosage. An endoscopic assessment at six months revealed complete mucosal healing. A parallel case was reported by the same authors, featuring a 35-year-old individual previously treated with adalimumab [38].

In summary, the collective evidence from multicentric studies, case series, and isolated reports suggests a potential role for tofacitinib in managing inflammatory pouch disorders. Indeed, the role of tofacitinib in managing CARP/CADP warrants further exploration through dedicated clinical trials. In this regard, we await the results of a recently concluded trial (i.e., NCT04580277) to provide more insights. However, it is essential to note that this Phase II trial was conducted in only six patients. Therefore, there is a clear need for a Phase III study with a larger patient cohort to ensure a more robust and realistic assessment of the efficacy of tofacitinib in this particular setting.

**Figure 1:**
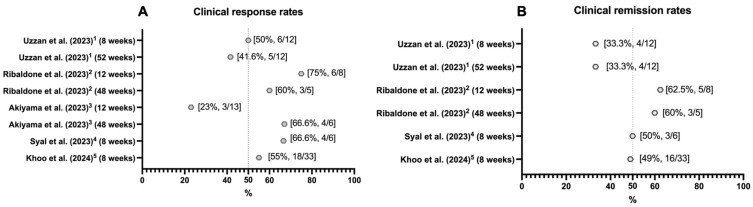
Rates of clinical response (**A**) and remission (**B**) reported in studies exploring the efficacy of tofacitinib in treating inflammatory pouch disorders. The data are expressed as a percentage rate and as the number of patients who achieved the outcome of interest (i.e., clinical response or remission) out of the total number of patients included in the analysis. The rate of 50% is indicated on the *x*-axis by a vertical dashed grey line. The superscripts related to each study explain the interpretation provided by each study regarding the rates of clinical response and clinical remission, and, specifically: (1) Clinical remission was defined as being steroid-free and antibiotic-free at week 8 (±4 weeks) and week 52 (±8 weeks) with a modified pouchitis disease activity index (mPDAI) <5 and, in cases where endoscopic control was lacking, as having an isolated clinical subscore <2. Clinical response was defined at week 8 (±4 weeks) and week 52 (±8 weeks) as being steroid-free with a reduction in mPDAI >4 at week 8 compared to baseline, with a reduction in mPDAI >1. In cases where endoscopic assessment was lacking, clinical response was indicated by a clinical subscore >1 at week 8 with a reduction of at least 1 point from the baseline score. (2) Clinical response at three months was defined as steroid- and antibiotic-free with a reduction in mPDAI of at least one point from baseline. Clinical response was defined at twelve months as a reduction in mPDAI of at least two points compared to baseline. Remission, both at three months and twelve months, was defined as an mPDAI score of 0. (3) Clinical response, assessed at month 3 (±2 months) and month 12 (±2 months), was evaluated as an improvement of at least two points from the baseline mPDAI. (4) Clinical response at 8 weeks was defined as a reduction of at least two points in the mPDAI compared to baseline with at least a one-point reduction in the endoscopic subscore. Clinical remission was defined as an mPDAI <5 compared to baseline with a reduction of at least two points compared to baseline and changes in clinical and endoscopic scores of the PDAI subscores, faecal calprotectin, and the 10-point Cleveland global quality of life scores. (5) Clinical response was defined as a reduction of at least two points in the clinical PDAI compared to baseline at week 8. Clinical remission was defined as an mPDAI <5 compared to baseline. The references related to the studies shown in the figure are as follows: Uzzan et al. (2023) [31], Ribaldone et al. (2023) [32], Akiyama et al. (2023) [33], Syal et al. (2023) [34], Khoo et al. (2024) [35].

As evident from Figure 1, which summarizes the rates of clinical remission/response identified across all conducted studies, there is a clear issue regarding the sample size, the heterogeneity of definitions for clinical response, the remission among the studies conducted (despite the global adoption of the PDAI score), and the significant variability in the recorded rates.

### 4.2. Upadacitinib

Upadacitinib is a JAK inhibitor and, as such, it reduces the signal transduction pathway induced by members of the JAK family (specifically JAK1). This occurs due to interactions with the downstream transcription factors of the signal transducers and activators of the transcription (STAT) family, which control a broad spectrum of downstream pro-inflammatory cytokines [20,24].

Upadacitinib is currently one of the leading treatments in the management of UC. In network meta-analysis, its effectiveness, measured by clinical response, clinical remission, and endoscopic improvement, has recently achieved a surface under the cumulative ranking curve of 99% at a dosage of 45 mg, both in the population naïve to advanced treatments and in the bio-experienced one [39]. Nevertheless, it has also demonstrated a remarkable speed in inducing early clinical remission within four weeks of treatment [40].

However, based on the available evidence (which is unfortunately limited to the case series), this performance profile has not automatically translated to pouch disorders. From the reported case series, the performance of this selective JAK_1_ inhibitor varies significantly and is, overall, not highly satisfactory (while awaiting dedicated randomized trials).

Ribaldone et al. [32] delineated, in a case series, six cases of upadacitinib treatment for CARP (of which five were males) with an average age of 37.5 years. Patients had undergone prior biological therapies before initiating upadacitinib, predominantly vedolizumab in most cases. At three months of follow-up, a clinical response was observed in only two out of six cases (33.3%), and an absolute antibiotic-free clinical remission was achieved in a solitary case (16.7%).

Lan et al. [41] compiled data within a six-patient cohort. The average age of the patients was 42.5 years, all of whom were non-smoker males with a precise diagnosis of UC prior to colectomy, all undergoing the creation of a J-pouch. In the majority of cases, they suffered from CARP (in one case, cuffitis). In the remaining three cases, it was a Crohn’s-like disease of the pouch. Almost all of them (except one patient) had previously undergone therapy with anti-TNF agents (predominantly adalimumab), and in four cases, they were also exposed to anti-interleukins (i.e., ustekinumab) and an equal number to anti-integrins (i.e., vedolizumab).

Interestingly, three of them had also received tofacitinib before the use of upadacitinib. None of the patients had undergone topical therapy with 5-ASA or budesonide therapy. The safety profile was optimal in all six patients without adverse events. Regarding effectiveness, however, the authors noted the absence of significant clinical and endoscopic improvements (except for improvements in extra-intestinal manifestations, largely articular) after six weeks of treatment.

Two subsequent case reports highlighted the successful treatment of chronic pouchitis with upadacitinib in the presence of concurrent duodenitis [42] and pyoderma gangrenosum [43].

As evident from Table 1, summarizing the relevant reported cases of treating inflammatory pouch disorders with upadacitinib, this particular treatment was employed in a predictable multi-failure setting to biologics, occurring both prior to the baseline IPAA indicated for UC and preceding biologics or small molecules indicated for targeted therapy of CARP or Crohn’s-like disorders of the pouch.

This makes it challenging to confidently assess the actual inhibitor’s effectiveness (or lack thereof) of this JAK1-selective inhibitor, especially in patients who are particularly complex to treat. In these case series, the rate of patients demonstrating a real and tangible clinical response, antibiotic- and steroid-free, is dramatically variable and not entirely satisfactory. Therefore, it is imperative to pursue further investigation through dedicated clinical trials.

### 4.3. Filgotinib

The evidence regarding the use of filgotinib in pouch disorders is, unfortunately, as of today, solely anecdotal. There is only one reported case of a 33-year-old male patient who was treatment-naive for advanced pouch-related treatment 17 years after the creation of the pouch with IPAA (although he was previously treated, before the procedure, with infliximab, vedolizumab, and ustekinumab). However, in this case, the patient did not achieve a clinical response even after three months into the treatment, with endoscopic deterioration measured by PDAI increased from a baseline of five to six in the three months [32].

## 5. S1P-R Modulators

Ozanimod is a high-affinity modulator for S1P-R1 and S1P-R2 receptors [44]. The mechanism of action is based on reducing T lymphocyte traffic in lymph nodes by inhibiting the interaction of S1P with S1PR1, ultimately reducing circulating lymphocytes, especially those expressing the chemokine receptor 7 [45]. Simultaneously, ozanimod allows for the reversible reduction in peripheral lymphocytes by facilitating the internalization and subsequent degradation of S1P1R [44]. Ozanimod was the second small molecule (following tofacitinib) to receive approval from the FDA for treating UC [45].

Currently, no available observational or interventional studies (such as trials) specifically address using S1P-R modulators in inflammatory pouch disorders in UC patients undergoing IPAA. Currently, the highest level of evidence is discernible in two reported cases within a broader case series [32].

The results of these two reports vary. In detail, the first described patient is a 34-year-old female with an IPAA for over ten years, whose UC had previously been treated with infliximab. The pouchitis (of the CARP type) was then subjected to ozanimod treatment after failure with multiple therapies, both conventional (5-ASA and budesonide) and advanced (including infliximab, vedolizumab, ustekinumab, and even tofacitinib), yet without achieving clear clinical benefit.

On the contrary, the second case involves a 24-year-old male with a recent placement of IPAA (for four years). Despite undergoing multiple prior treatments for UC (with infliximab, adalimumab, vedolizumab, and filgotinib) at a young age and multiple treatments for CARP with vedolizumab and ustekinumab, he achieved a clinical response at three months, free from steroids and antibiotics. Nevertheless, an evident clinical remission was not achieved during the same time frame.

All of this would lead us to withhold a definitive judgment on the effectiveness of ozanimod in this setting and await more robust evidence for a genuine benefit. Despite this, some guidelines support its use in CARP and CADP [10].

## 6. Current Issues and Unmet Needs in Managing Inflammatory Pouch Disorders with Biologics and Small Molecules

When indicated, constructing an IPAA after proctocolectomy is the definitive treatment for UC. Despite its significant advantages in the disease activity control in UC, it presents considerable challenges. Among the most frequent long-term adverse outcomes of IPAA, inflammatory disorders of the pouch, particularly pouchitis (which dominates in over 29% of cases), along with defecatory dysfunction, primarily fecal incontinence (with an estimated incidence of 21%), are notable [46]. Without the appropriate treatment, these issues can expose individuals to the risk of pouch failure and the need for re-intervention for its removal. All of this suggests the necessity of creating a pouch in high-volume centers with a certain level of experience in this surgery [47], where expertise is crucial not only in the surgical procedure but also in managing post-surgical complications, such as leakages. This approach increases the likelihood of successful salvage surgery for the pouch [48].

The treatment of chronic pouchitis has historically been approached with antibiotics, often in a cyclic combination. Among these, ciprofloxacin, metronidazole, tinidazole, and rifaximin have been the most commonly used, along with steroid treatments such as budesonide or oral beclomethasone [49]. Regarding topical therapy (i.e., enema) with budesonide, a comparative trial in active pouchitis is available, which demonstrated greater tolerability and comparable efficacy when compared to metronidazole [50].

The era of biologics has certainly offered greater treatment possibilities and has integrated into the pouch’s management of CARP, CADP, and Crohn’s-like disease. A recent meta-analysis [51] estimated the clinical response of patients with chronic pouchitis treated with infliximab at 51%, adalimumab at 47%, and ustekinumab and vedolizumab at 41%, providing the interpretation that all these treatments are conceivable in this category of patients, as also suggested by the available guidelines [10,49,52]. Among these, infliximab has retained particular importance due to the availability of more evidence suggesting its superior performance compared to adalimumab [53]. In contrast, as previously mentioned, vedolizumab is the only biologic authorized for pouchitis and has a robust clinical trial supporting its efficacy [11]. Although a direct comparison is not entirely feasible due to the significant disparity in data and evidence for inflammatory disorders of the pouch between monoclonal antibodies and small molecules, the rates derived from the series (Figure 1, Table 1), particularly for JAK inhibitors where more data are available (with very few published cases on S1P-R modulators), provide encouraging results. This is especially true for pan-JAK inhibitors (tofacitinib), which show more promise than JAK1-selective inhibitors such as upadacitinib, as the latter has already produced heterogeneous data in case reports (Table 1).

However, the design and implementation of ad hoc non-IBD-derived trials for new pharmacological mechanisms targeting pouch inflammatory disorders do not progress at the same pace as those devoted to pre-IPAA IBD. Consequently, pouchitis-directed dedicated trials are few and do not always have positive results. For example, a trial on fecal microbiota transplantation for chronic pouchitis has already been conducted, but it did not yield positive results [54]. Nonetheless, the use of probiotics (such as *Lactobacillus plantarum* 299 plus *Bifidobacterium* Cure21 and *Clostridium butyricum* MIYAIRI) has produced heterogeneous results in dedicated trials and, in general, has not definitively demonstrated explicit control over the inflammatory pathogenesis [55,56]. Conversely, probiotics might offer potential benefits when added to conventional treatment (with 5-ASA or immunosuppressants), as seen in the trial with VSL#3 [57]. Somatostatin analogues, such as octreotide, have yielded disappointing results in a small trial involving patients with IPAA [58]. A few additional trials have focused on reducing diarrheal symptoms in pouchitis, for example, with spherical carbon adsorbent [59], rifaximin [60], or bismuth carbomer foam enemas [61], which have shown some benefit in this regard. Nonetheless, several trials are currently ongoing, including those on fecal microbiota transplantation (NCT05829109), new antibiotic formulations based on fosfomycin and metronidazole (NCT04979832), ursodeoxycholic acid (NCT03724175), and live biotherapeutics such as EXE-346 (NCT05938465).

This review highlights that, although preliminary, various pieces of evidence are available (albeit not of high quality due to the nature of non-randomized study designs, represented mainly by case series) regarding the use of JAK inhibitors (primarily tofacitinib and upadacitinib) in the treatment of CARP and CADP and, to a lesser extent, in the treatment of Crohn’s-like disease of the pouch. On the other hand, reports on filgotinib and ozanimod and information on etrasimod are still strongly pioneering or absent, respectively. So, the major challenge in addressing inflammatory disorders of the pouch (already inherently complex to treat) is to establish treatments that, for the most part, lack robust evidence. Currently, there is significant difficulty in formulating guidelines supported by high-strength recommendations using standardized methodologies such as GRADE [62].

However, the availability of preliminary evidence provides an excellent opportunity to consider their use under close clinical scrutiny and the appropriate assessment of the risk–benefit balance in patients with refractory, multi-failing forms of pouchitis. Nevertheless, in a significant portion of cases reported in the literature, small molecules have been used in the settings of multi-failure of the previous advanced therapies (including biologics and other small molecules), both in the pre-IPAA setting and the context of chronic pouch disorders. This responds to the urgent need to prevent pouch failure in hard-to-treat patients who have failed multiple biologics, for which more robust evidence is already available.

## 7. Conclusions

In conclusion, this review examined the therapeutic potential of small molecules in addressing the pouch’s chronic inflammatory disorders in patients undergoing IPAA for UC. This setting poses a significant challenge in its management. The multifaceted approach to treatment involves a spectrum of interventions, ranging from conventional therapies to advanced biologics and, most recently, small molecules. Rigorous, well-designed clinical trials are imperative to establish the efficacy, safety, and long-term outcomes of small molecules in treating chronic inflammatory disorders of the pouch.

At this point, while awaiting randomized controlled trials on small molecules, real-life experiences must necessarily supplement the need for ongoing evidence on using these molecules in the context of post-IPAA inflammatory pouch disorders. Consequently, it is conceivable and desirable that study groups and international IBD-focused societies promote continuous education on the therapy of these disorders in line with literature updates. Conversely, creating real-world registries for collecting data on the effectiveness and safety of advanced treatments used in pouch-related conditions should also be encouraged.

## Figures and Tables

**Table 1:** Clinical and demographic characteristics of reported cases of treatment with upadacitinib in instances of chronic antibiotic-refractory pouchitis.

Sex	Age	IPAA Duration (Years)	Pouch Anatomy	Previous 5-ASA/Budesonide/Steroids Pouchitis Treatment	Previous Pouchitis Advanced Treatment	Previous UC Advanced Treatment	Clinical Response	Ref.
M	41	5	N/A	No	VDZ	No	No ^a^	[32]
F	21	4	N/A	No	IFX	IFX, ADA, VDZ	No ^a^	[32]
M	38	15	N/A	No	ADA, VDZ	IFX, ADA	No ^a^	[32]
M	23	4	N/A	No	VDZ, UST, OZA	IFX, ADA, VDZ, FIL	Yes ^a^	[32]
M	50	4	N/A	No	ADA, VDZ	IFX, ADA, VDZ, FIL	No ^a^	[32]
M	52	4	N/A	No	UST	IFX, ADA, VDZ, TOFA	Yes ^a^	[32]
M ^b^	23	4	J-pouch	5-ASA, BUD	ADA, VDZ	N/A	No	[41]
M ^c^	59	15	J-pouch	BUD	ADA, UST, VDZ, TOFA	N/A	No	[41]
M ^c^	71	28	J-pouch	No	ADA, UST, TOFA	N/A	Yes ^d^	[41]
M	59	23	J-pouch	BUD	IFX, ADA, UST, VDZ, TOFA	N/A	No	[41]
M	24	13	J-pouch	Prednisone	ADA, VDZ	N/A	No ^e^	[41]
M ^f^	19	3	J-pouch	5-ASA, BUD	UST	N/A	No ^f^	[41]
M	27	1	N/A	Prednisone (oral and enema)	None	IFX, UST, VDZ, tacrolimus	Yes ^g^	[42]
F	40	N/A ^h^	J-pouch	N/A	UST	IFX	Yes ^h^	[43]

Acronyms: M: male; F: female; N/A: not available; IPAA: ileal pouch-anal anastomosis; UC: ulcerative colitis; 5-ASA: 5-aminosalicylic acid; BUD: budesonide; IFX: infliximab; ADA: adalimumab; VDZ: vedolizumab; UST: ustekinumab; TOFA: tofacitinib; FIL: filgotinib; OZA: ozanimod. ^a^ The clinical response was conclusively characterized as steroid- and antibiotic-free, along with a reduction of at least 1 point from the baseline of the modified pouchitis disease activity index (3 months). ^b^ In this case, the indication for upadacitinib treatment was given for Crohn’s disease and cuffitis in the pouch. ^c^ In this case, the indication for upadacitinib treatment was given for Crohn’s disease of the pouch. ^d^ The response was conclusively described as nonspecific, characterized by a mild improvement in symptoms after 12 weeks of upadacitinib treatment and a slight endoscopic improvement observed at pouchoscopy eight weeks into the treatment. ^e^ Despite the absence of a declared clinical improvement, a modest improvement in endoscopic inflammation is reported at week eight from the initiation of treatment with upadacitinib. ^f^ In this case, upadacitinib treatment was used concurrently with azathioprine, the latter indicated for concomitant autoimmune hepatitis. ^g^ The patient achieved a modified pouchitis disease activity index of 0 on endoscopic evaluation 9 months after starting UPA and remained in sustained remission for the following year. ^h^ In this case, the precise timing of the duration of IPAA is not indicated, and it is reported that after 6 months of treatment with UPA, there was an unspecified improvement in pouchitis.
